# Influence of pathogens on host genome and epigenome in development of head and neck cancer

**DOI:** 10.1002/cnr2.1846

**Published:** 2023-06-15

**Authors:** Sanket Desai

**Affiliations:** ^1^ Independent Researcher

**Keywords:** cancer associated pathogens, head and neck cancer, host‐pathogen interaction

## Abstract

**Background:**

Head and neck cancer (HNSCC) is a heterogeneous group of cancers, affecting multiple regions such as oral cavity, pharynx, larynx, and nasal region, each showing a distinct molecular profile. HNSCC accounts for more than 6 million cases worldwide, soaring mainly in the developing countries.

**Recent findings:**

The aetiology of HNSCC is complex and multifactorial, involving both genetic and environmental factors. The critical role of microbiome, which includes bacteria, viruses, and fungi, is under spotlight due to the recent reports on their contribution in the development and progression of HNSCC. This review focuses on the effect of opportunistic pathogens on the host genome and epigenome, which contributes to the disease progression. Drawing parallels from the host‐pathogen interactions observed in other tumour types arising from the epithelial tissue such as colorectal cancer, the review also calls attention to the potential explorations of the role of pathogens in HNSCC biology and discusses the clinical implications of microbiome research in detection and treatment of HNSCC.

**Conclusion:**

Our understanding of the genomic effects of the microbes on the disease progression and the mechanistic insights of the host‐pathogen interaction will pave way to novel treatment and preventive approaches in HNSCC.

## 1 INTRODUCTION

Head and neck cancer (HNSCC) is a complex set of diseases, mainly caused due to exposure to etiological factors, including the carcinogens derived from the metabolism of tobacco, smoking, and alcohol. The disease accounts for new 660 000 cases and 325 000 deaths annually.^1^ Occult node metastases and poor overall 5‐year survival rate after advanced treatment with chemo‐ and radiotherapy modalities remain the major challenges in the HNSCC. Over 65% of the HNSCC cases are attributable to the tobacco use, smoking and alcohol consumption.^2^ The HNSCC genome undergoes several insults due to the exposure to diverse endogenous and exogenous etiological factors. Comprehensive genomic analysis has identified several molecular subtypes based on mutation, copy number alteration, gene expression and change in methylation at cancer gene loci.^3^ The analysis identified a distinct genomic subtype associated with human papilloma virus (HPV) infection, possessing specific mutation profile (detailed later). Smoking‐related HNSCCs demonstrate near universal loss‐of‐function *TP53* mutations, inactivation of *CDKN2A* by copy loss and frequent copy number alterations including amplification of 3q26/28 and 11q13/22. The peculiar genomic characteristics also include loss‐of‐function alterations of the chromatin modifier *NSD1*, WNT pathway genes *AJUBA* and *FAT1*, and activation of oxidative stress factor *NFE2L2*, mainly in laryngeal tumours.^3^ Ethnic and site‐specific HNSCC cancer genome profiles and variability in the frequency of the common cancer genes is also described in other studies.^4^, ^5^ Genomic characterisation has resulted in discovery many therapeutically relevant alterations, however additional risk‐factors remain to be discovered as the HNSCC cases are on the rise in the developing countries, which do not associate with the known etiological factors. In recent years, additional risk factors have emerged in the form of pathogenic microbes, which influence the genesis and progression of many different cancers. Global estimates suggest an approximate cancer attribution of 15%–20% cases to microbes,^6^ with a burden of nearly 2.2 million infection associated cases in the 2018 alone.^7^ Majority of the pathogens associated with cancer are oncogenic viruses including human papilloma virus (HPV), Epstein–Barr virus (EBV), Human T‐lymphotropic virus 1 (HTLV1), Kaposi sarcoma‐associated herpesvirus (KSHV), hepatitis B (HBV) and hepatitis C virus (HCV).^8^ Among bacteria, oncogenicity in animal model systems has been established for *Helicobacter pylori*.^7^, ^8^ Other reported cancer‐pathogen associations include, *Chlamydia pneumonia* with lung cancer,^9^ Salmonella typhi infection with gallbladder cancer^10^, ^11^ and *Streptococcus bovis*, genotoxic *Escherichia coli*, enterotoxigenic *Bacteroides fragilis* and *Fusobacterium nucleatum* with colon cancer.^12^, ^13^, ^14^, ^15^, ^16^ Head and neck cancer is no exception to this. In fact, oral cavity being the most exposed sites in the human body, acts a gateway constantly interfacing with the microbes. The constant exposure of the microbial species is through the fluids we drink, the food we ingest and in the nasopharyngeal area via the air we breathe. Hence, microbes colonising any part of oral cavity tend to spread to the neighbouring epithelial surfaces in the head and neck region, passively helped by the fluid phase of saliva in the subgingival and gingival region. Opportunistic pathogens among the colonisers, causing dysbiosis in the oral microbiota, cause several diseases including periodontal disease, dental caries, candidiasis, and others.^17^ Systemic and chronic inflammation associated diseases such as rheumatoid arthritis,^18^ Alzheimer's,^19^ hypertension,^20^ systemic lupus erythematous (SLE)^21^ and cancers, including colorectal,^22^ pancreatic,^23^ oesophageal,^24^ lung^25^, ^26^ and head and neck,^27^ have been associated with oral microbes. The overall genetic material of the microbes in the oral cavity is termed as oral microbiome, which consist of wide range of species of viruses, archaea, fungi and over 700 species of bacteria. The Human microbiome project^28^ aimed to provide a comprehensive catalogue of microbes across different human tissues and establishment of a ‘core microbiome’ for every niche in the healthy humans tissue, if any. To assess the possibility of establishing a core microbiome for the oral cavity (a subset of head and neck), Caselli E., et al.^29^ studied the microbiome variation among 20 healthy individual using whole genome sequencing (WGS). This study concluded that the composition of oral microbiome remained largely conserved, with no statistically different microbial ratios, providing a ‘eubiosis’ reference. Diverging from the normalcy, a dysbiosis state specifically caused by enrichment of some pathogenic strains may result into chronic inflammation state, assisting the tumorigenesis process. Different sites of head and neck region are no exception to this.

## 2 MICROBES ASSOCIATED WITH HEAD AND NECK SQUAMOUS CELL CARCINOMA

Many cancer‐associated pathogens are involved in development of HNSCC. The most well studied and established association is the HPV infection in oropharyngeal cancer. Systematic genomic analysis of the HNSCC tumours have identified the HPV‐positive tumours to be having a distinct pattern, with lower frequency alterations observed in the tumour suppressor genes, as compared to the HPV negative sub‐type.^3^ The HPV positive tumours form a distinct clinical and molecular sub‐type, with having better prognosis and higher overall survival.^30^ The detailed molecular mechanism of the HPV‐induced carcinogenesis, via inactivation of p53 and pRb, has been detailed in the following review.^31^ Clinically relevant stratification of the HNSCC tumours based on pathogens, like HPV provide an avenue to design therapeutic and preventive strategies. Other oncogenic viruses, such as HCV, EBV and HBV, have also been reported to cause site specific HNSCC.^32^, ^33^, ^34^ EBV also forms a distinct sub‐type with site specific infection in nasopharynx, discussed later in the review. Expression of latent EBV genes such as Epstein–Barr nuclear antigen 1 (*EBNA1*) and Latent Membrane Protein 1 (*LIMP1*), interfere with core cellular machinery and cell cycle genes in nasopharyngeal carcinoma (NPC).^35^ As with many different cancer types, such as cervical and liver cancer, viral carcinogenesis has been studied at molecular and pathway resolution in HNSCC.

In recent years, application of genome and metagenome (16S RNA) sequencing has revealed the complex composition of the microbiome across various sites in the head and neck region and their association with cancer.^27^ Several bacterial species found in different sites of head and neck region are potentially flagged as cancer causing. Abundance of bacteria like, *Porphyromonas gingivalis*, *Fusobacterium nucleatum*, *Streptococcus*, *Prevotella*, *Veillonella*, *Actinomycetes*, *Bacteroides*, *Clostridium*, *Klebsiella*, *Citrobacter*, *Streptococcus*, *Enterobacter* and *Serratia*, have been found to be abundant or enriched in head and neck cancer^36^, ^37^, ^38^ Each of these bacteria are independently known to induce inflammation in the state of dysbiosis. Site specific cancer‐pathogen associations have also been identified for the above‐mentioned bacteria, including *Fusobacterium, Streptococcus, Prevotella* with laryngeal,^39^ *Fusobacterium* with oral cavity and tongue tumours,^40^, ^41^, ^42^ *Porphyromonas gingivalis* with oral cancer,^43^ are some of the examples. Among the mentioned bacteria, *Porphyromonas gingivalis* and *Fusobacterium nucleatum* are reported to promote tumour progression in‐vivo, in the oral‐specific chemical carcinogenesis mode.^44^ Mechanistically, the lipopolysaccharide in the outer membrane of these Gram‐negative bacteria interacts with Toll‐like receptor 4 (*TLR4*), which leads to downstream activation of Myeloid differentiation primary response 88 (*MyD88*) dependent and MyD88‐independent pathways, which then trigger the nuclear factor κB pathway leading to the release of pro‐inflammatory cytokines.^45^, ^46^ Action of such bacteria in the tumour microenvironment are primarily driven by heightening of the innate immunity against them.^47^ *Streptococcus* and *Prevotella* have also been postulated to have similar effect on the change in cytokine profile and to activate of innate immunity.^36^ *Porphyromonas gingivalis* is also reported to increase the invasiveness of the oral cancer cells, via induction and activation of pro‐MMP9.^48^ Similar correlation is also observed in abundance of *Fusobacterium* and expression of *MMP10* in tongue cancer,^40^ a biomarker of lymph node metastases mechanistically shown to be driving invasion and migration in tongue cancer.^49^ Details of the mechanistic involvement of the 2 gram‐negative opportunistic pathogens with oral cancer has been reviewed elsewhere.^50^, ^51^ As bacteria remain the dominant species of the metagenome, the association analysis warrants a careful inspection and requires supporting biological assessment of the role of cancer associated pathogens in the cancer microenvironment.

**FIGURE 1:**
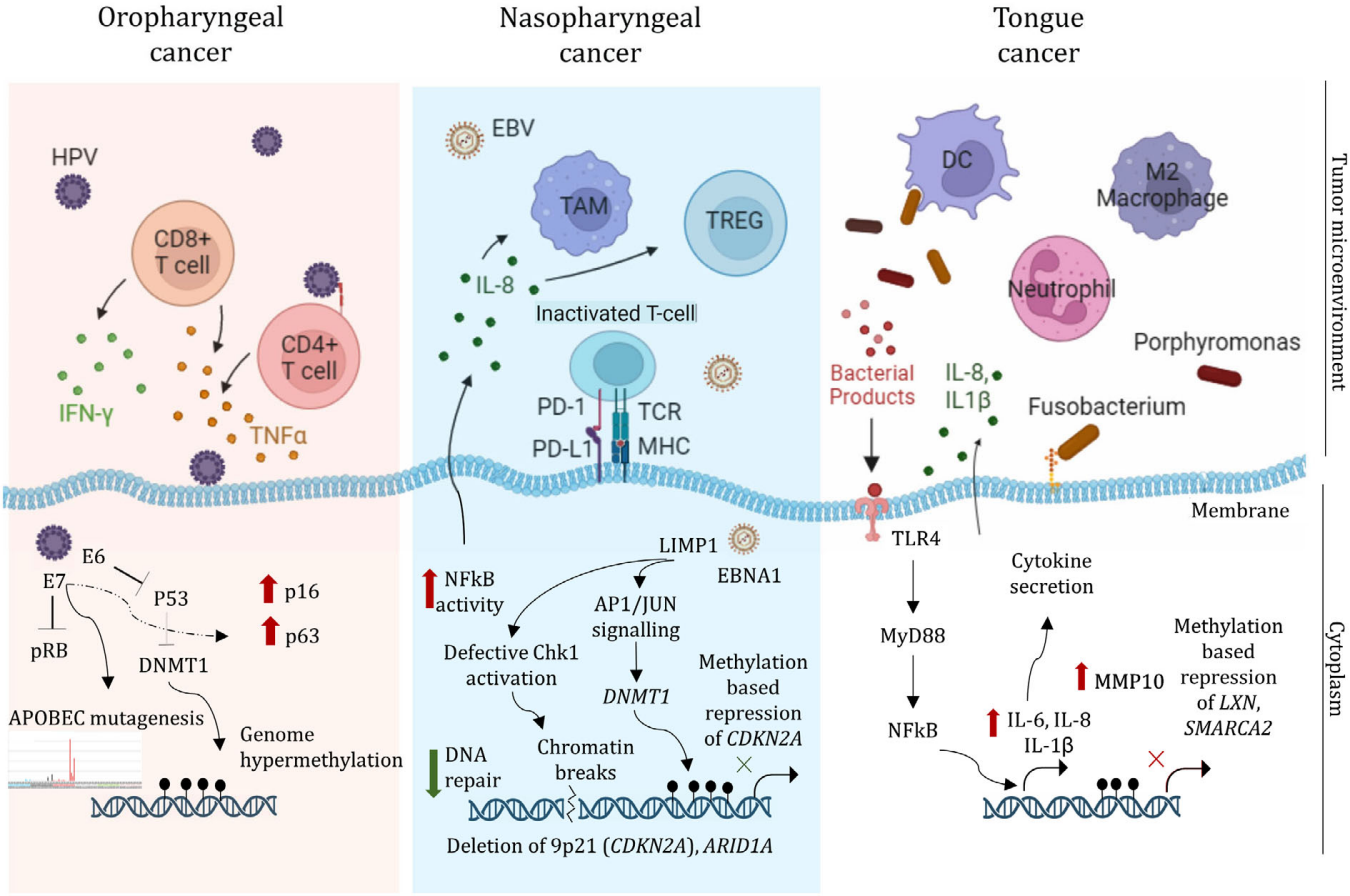
Genomic and epigenomic influence of the pathogens in distinct anatomical sub‐sites of HNSCC. Illustration depicts the immune cell profile in the tumour micro‐environment consisting of predominant cell types such as T cells (CD4+, CD8+ in HPV positive subtype), tumour associated macrophages (TAM), regulatory T cells (TREG) (in EBV +ve subtype) and innate immune cell enrichment – dendritic cells (DCs), neutrophils and M2 macrophages (in *Fusobacterium* enriched tongue tumours). Inside the cytoplasm, pathogenic particles are mentioned which directly or indirectly interact with the host cellular machinery causing different types of genomic aberrations. The up‐ red arrows indicate increase in activity or expression and the down‐ green arrows indicate the decrease in activity. Black circular flags on the genome represent methylation.

The field of microbiome analysis in cancer research is presented with multiple challenges. Including biases due to sensitivities of different detection methods, microbial contamination in the process of tumour sample processing, such as clinical acquisition of sample, collection and processing, quality control and quantification. Also, oral cavity and its sub‐sites being the most exposed regions of the human body, environment, food, and habits may also alter the composition greatly, adding another layer of microbial composition dynamics, which may be only identified and corrected in a large scale study of patient samples.^52^ Moreover, the variation observed in the microbial makeup in the HNSCC sub‐sites may yield conflicting outcomes regarding potential involvement of an opportunistic pathogen in cancer prognosis.^51^ However, detailed mechanism‐based studies involving the bacterial species in other cancer types, including colorectal and oesophageal, provide clues about the involvement of these microbes in other epithelial squamous cell carcinomas such as oral cancer. Although, primary focus of investigation largely remains to be association of viral and bacterial pathogens with HNSCC, fungal species of genus *Candida*, have also been associated with treatment induced complications as well as carcinogenesis.^53^ A recent pan‐cancer analysis has revealed a direct or indirect (via interactions with bacterial colonies) role of fungal species in cancer progression across several different cancer types.^54^ In overall, a complex conjunction of the microbial abundances and presence of specific pathogenic species in the tumour microenvironment determine the fate of the disease progression. A compilation of studies characterising the microbiome, its function and implications across various tumour types including the HNSCC has been provided in a review.^55^ Another recent review provides an exhaustive compilation the clinical studies investigating HNSCC associated microbes, their tumour stage specific presence and their relation to prognostic outcomes.^56^ The measurable molecular effects of the dysbiosis of these pathogenic microbes can be seen in form of genomic, epigenomic or immune cell composition derived signatures. These are further discussed in the following sections and summarised in Figure 1.

## 3 DIRECT IMPACT OF MICROBES ON THE HNSCC GENOME

Mutational profile of the HNSCC tumours show differential alterations in the genome, which correlate to the etiological factors and anatomical subsites. Analysis of landscape of genomic alterations provided a molecular classification for HNSCC.^3^ Among the broad class of tumours related to smoking, loss of function mutations in the tumour suppressor gene *TP53* and deep deletion of *CDKN2A* gene copy, are the commonly observed alterations. A distinct sub‐class of oral tumours harbour activating mutations of *HRAS* or *PIK3CA*, and inactivating mutations in *CASP8*, *NOTCH1* genes. On the other hand, majority of the laryngeal tumours harboured alterations in *Wnt* pathway genes, a chromatin modifier (*NSD1*) and activation of *NFE2L2*, an oxidative stress factor. As with the exposure to environmental factors, genomic alterations are also defined by pathogenic infection in HSNC.^57^ The incidence of HPV associated with HNSCC varies across the anatomical sites and geographical locations (populations), which creates a difference in the global genomic alteration profile of the HNSCC tumours. HPV proteins E6 and E7, inactivate P53 and RB protein, respectively, which is causally linked to malignancy.^58^ Because the deactivation of the tumour suppressor genes in the HPV positive tumours takes place due to viral activity, this sub‐group of tumours harbour lower mutations in *TP53* and *CDKN2A* genes at early stages and instead a higher mutation abundance is found in the *PIK3CA*, *E2F1*, and *TRAF3* genes.^3^ Hence, the overall progression of genome evolution of the HPV positive tumours follows a different trajectory. Tumours positive for HPV are also known to undergo apolipoprotein B mRNA‐editing catalytic polypeptide‐like (APOBEC) mediated mutations,^59^, ^60^ which causes well‐known characteristic endogenous mutational signature. Further, integration of HPV in human genome is commonly observed in both cervical and HNSCC tumours. Integration of HPV into the human genome, has been found to be enriched in the HNSCC associated genes, as well as genomic loci (such as *CD274*, official gene name for *PD‐L1*) important for immune function, ultimately correlating with survival outcomes of the patients.^61^ Additional molecular features altered due to HPV prevalence in HNSCC are reviewed elsewhere.^33^

In contradiction to the HPV infected HNSCC patient, recurrent somatic copy number changes are observed in EBV infected nasopharyngeal carcinoma (NPC) patients. Most of the EBV infected NPC patients harbour frequent deletion of 9p21 chromosomal region containing *CDKN2A* gene.^62^ In the EBV enriched sub‐group of NPC patients, additional copy number variations were also observed in well‐known cancer genes including, *CCND1*, *AKT2*, *MYC*, *TP53*, and frequent deletions in an important component of the SWI/SNF complex, *ARID1A*. EBV infected tumours are also reported to have a distinct mutational signature not contributed by endogenous *APOBEC3*‐induced mutagenesis, unlike the HPV positive sub‐group of patients.^62^ Furthermore, EBV viral gene activity has been reported to results in chromosomal instability in NPC. The *LIMP1* viral gene impairs the G2 checkpoint in human NPC cells through defective Chk1 activation, leading to unrepaired chromatin breaks.^63^ EBV infection also causes reduced DNA damage repair responsiveness, by controlling expression of mismatch repair (MMR) and base excision repair (BER) pathway members, including *MLH1*.^64^ Although the genome alteration landscape of EBV and HPV positive HNSCC patients vary greatly, immunotherapy and therapeutic vaccination is a proposed treatment option due to similarity in the immune activity profiles of the two sub‐groups.^65^, ^66^

Sparse experimental evidence currently exists for activity of bacterial strains in altering HNSCC genomes. However, effect of bacterial interaction with epithelium is reported to have direct implications on the DNA stability and mutation distributions. The classical example is of *Helicobacter pylori* which is known to induce genomic instability in gastric cancer cells. The association of *H. pylori* with the decrease in the DNA repair ability, generation of mutator phenotype and mutation induction in mitochondrial genes in gastric cancer, provides a framework to study genome damaging effects of a bacteria (reviewed here^67^). Multiple studies in recent years have identified role of other bacterial metabolites in the genomic instability, increase in mutation burden and its distribution across the colon cancer genome. *Escherichia coli* is known to induce DNA double strand breaks in the eukaryotic cells.^68^ Building on this study, Pleguezuelos‐Manzano C., et al., identified a mutation signature induced by colibactin – a protein encoded by *PKS* genes, produced by the pks + *E. coli*.^68^ The study also highlights the effect of this mutagenesis at specific gene loci, including genes like APC, which are the founder mutations for the colon tumorigenesis. Also, Bacteroides fragilis toxin (BFT) has been reported to up‐regulate Spermine oxidase (SMO), a polyamine catabolic enzyme, which increases the DNA damage via reactive oxygen species (ROS).^69^ *Bacteroides fragilis* along with *Fusobacterium nucleatum* – a known colorectal cancer associated *oncobacterium*,^13^ show significant enrichment in the tumours deficient for DNA mismatch repair in colon tumours.^70^ Another study suggests correlation of microsatellite alterations and DNA damage with *Fusobacterium* infection in colon cancer.^71^ Since gastric and colon tumours are extensively characterised with respect to the microbiome, we find pronounced evidence of effect of bacterium and their metabolites on the genomes of these tissue types. However, the studies also highlight characteristics of the individual microbes in disease biology and details of their interaction with the human genome. Among the mentioned colon associated bacterium, *Fusobacterium nucleatum* has been reported to form a distinct subgroup of HNSC, exclusive to HPV.^40^ The *Fusobacterium* enriched tumours behave clinically different, having increased extracapsular spread and higher inflammation. In vitro and in‐vivo, studies also provide strong validation for the *Fusobacterium* induced inflammation.^44^, ^72^ Extensive evidence of direct effect of *Fusobacterium* on the DNA in HNSCC may be lacking, but an indirect inflammation induced host‐genome damaging role of the opportunistic pathogen cannot be ruled out.^73^ Also, preliminary experimental evidence of *Fusobacterium* induced DNA damage in oral cancer exists,^74^ and it needs further validation across different experimental systems in‐vitro and in‐vivo.

## 4 MODULATION OF EPIGENOME BY MICROBES IN HNSCC

The behaviour of a cancer cell cannot be explained entirely based on its genetic makeup. Epigenome plays an equally important role in cancer progression. Epigenome includes the control of gene expression via DNA methylation on the CpG island regions near the gene promoter and the chromatin architecture. DNA methylation (both hyper‐ and hypo‐) affects HNSCC tumours differently at different stages of the disease. In the human cell, DNA methylation is catalysed by the DNA methyltransferase (DNMT) family, consisting of 3 members: *DNMT1*, *DNMT3a* and *DNMT3a*. In cancer, alteration of expression of DNMT members is well characterised.^75^ In the scheme of events leading to carcinogenesis, DNA methylation is one of the early events observed in the pre‐malignant tissue even before the onset of cancer. This hypothesis has also been validated in the HNSCC cell line model systems.^76^ An overview of the role of methylation in HNSCC disease progression is reviewed elsewhere.^77^ Since the methylation status of specific gene loci in a cell is robustly maintained, some of the initial studies also used marker gene–based status as a predictive marker for HNSCC.^78^ In a general scenario, a global hypomethylation prevails at the initiation of the tumour formation leading to the expression of the repetitive elements, which constitute ~50% of the genome. The global hypomethylation has been associated with smoking, alcohol consumption and disease stage,^79^ whereas HPV–associated HNSCC tumours are known to be hypermethylated. As DNA methylation is a technique used by the host cells to suppress expression of the invading viral particles, its aberrant execution leads to carcinogenesis.^80^ As with HPV in HNSCC,^3^ these virus–induced global methylation changes have also been captured for other viruses involved in several different cancer types, such as EBV in gastric cancer,^81^ HBV and HCV with hepatocellular carcinoma^82^ and HPV for cervical cancer.^83^ A strong CpG methylator phenotype (CIMP) is also a common feature of the EBV‐associated nasopharyngeal cancer as EBV may alter the activity of host DNA methyltransferase and demethylase to establish viral methylome.^35^ A specific example is loss of expression of p16 due to promoter hypermethylation, in EBV infected nasopharyngeal carcinoma samples.^84^ The latent EBV protein LIMP1 is also reported to upregulated *DNMT1* expression, via activation of AP‐1/JUN signalling. Promoter hypermethylation–based silencing of crucial cell regulatory proteins, such as *PTEN*, by LIMP1 has been reported in gastric carcinoma.^85^ Since the viral proteins target important hub proteins in human cells, like p53 in case of HPV infection or p16 in EBV infected squamous cells, their inactivation leads to a global change. For example, p53 negatively regulates DNMT1 by repressing its expression. However, p53 inactivation due to mutation or viral activity leads to over–expression and hypermethylation in HNSCC tumours.^86^

Recent studies suggest that the microbiome may modulate the host epigenome through production of metabolites, immune system modulation, and direct interaction with host cells. Preliminary assessment of the possible involvement of microbiota in methylation events has been assessed using animal model studies and they conclude that use of antibiotics against majority of primary gut colonisers affects the host methylation pattern.^87^ Gut microbiome, along with diet also influences the epigenome in cancer and other diseases. The changes in the epigenome are found to be specific to the species in question, such as *Lactobacillus acidophilus*, *Bifidobacterium infantis*, and *Klebsiella* species.^88^ There are multiple ways in which this effect takes place. One prominent effector molecule which creates a differential methylation effect is the bacterial metabolites, such as short‐chain fatty acids (SCFAs). Gut microbiome has been shown to produce SCFAs that can modulate histone acetylation and gene expression. SCFAs have also been shown to regulate immune cell function, which may indirectly affect the epigenome.^89^, ^90^ As a model system, colon tissue provides examples of pathogen–associated methylation changes which also act as a risk factor for cancer.^91^ An integrated analysis of metagenomic sequencing and DNA methylation has revealed that *Hungatella hathewayi* and *F. nucleatum* enrichment was associated with hypermethylation of tumour suppressor genes in colon cancer tissue.^92^ Specifically, *H. hathewayi* caused promoter hypermethylation of *SOX11*, *THBD*, *SFRP2*, *GATA5*, and *ESR1*, whereas *F. nucleatum* associated with methylation of *MTSS1*, *RBM38*, *PKD1*, and *PTPRT*. Promoterspecific methylation activity of microbes may assist the cancer cell at specific stages of development, also providing an explanation for the association of stage–specific dynamics of the bacterial population in tumour microenvironment. Interestingly, a recent study provides evidence that *Fusobacterium nucleatum* has the ability to regulate epitranscriptome. *KIF26B*, a member of the kinesin (KIF) family, is a known oncogene which promotes metastasis in gastric cancer and colorectal cancer.^93^, ^94^ *METTL3* is an m^6^A methyltransferase suppressing *KIF26B* via degradation. However, *F. nucleatum* via activation of YAP signalling and subsequent transcriptional regulation of *METTL3*, results in *KIF26B* reactivation and ultimately metastases of colorectal cancer cells.^95^ Although methylation plays a major role in HNSCC development, very little is known about effect of specific pathogen in modulating the same. A recent study by Chen Z., et al. identified association of enrichment of *Fusobacterium nucleatum* with the gene promoter methylation within host, particularly causing hypermethylation of tumour suppressor genes *LXN* and *SMARCA2*.^96^ Although this review does not detail effect of microbes on other epigenetic interactions in head and neck cancer, there exist sparse reports which point in that direction. In addition to affecting the methylation status on at specific loci in human genome, microbial interactions may also act via histone acetylation (chromatic states), as well as non‐coding RNA (ncRNA) expression modulation. The periodontal pathogens have direct impact on the histone acetylation status in oral mucosa.^97^ Several reported associations are catalogued in form of a database between the microbes and acetylation or regulatory ncRNA expression in different cancer types, including head and neck cancer.^98^ In overall, literature suggests that microbes greatly influence the epigenome of the host epithelial cancer cells. However, more investigations are required in understanding the breadth of effect of pathogens on epigenetics of HNSCC. Also, mechanistic studies and crossmodel system validations are need of hour in this area of research.

## 5 CLINICAL AND THERAPEUTIC RELEVANCE OF MICROBIOME IN HNSCC

The genomic and epigenomic characteristics imparted by various pathogens lead to phenotypic variability in the tumours. The precise mechanisms connecting molecular influences and clinical outcomes may require further systematic investigation. Nevertheless, microbiome research is on the verge of entering into mainstream clinical evaluation with several leads arising from the associations and impact of microbes on HNSCC. Microbial dysbiosis of the oral cavity has been previously proposed to be a primary screening tool in not only for oral cancer,^99^ but other cancer types including colorectal^100^ and pancreatic cancer.^101^ Moreover, the microbial dysbiosis is correlated with survival differences in HNSCC. Specifically, an abundance of *Fusobacterium nucleatum* and *Porphyromonas gingivilis* is clinically correlated with poor survival in HNSCC in a site‐specific manner.^40^, ^102^ Poor survival outcomes with *Fusobacterium* abundance has been also previously reported in colorectal cancer, wherein the authors proposed development of preventive and treatment strategies targeting intestinal microflora by probiotics and antibiotics.^103^ Use of antibiotics in colorectal adenocarcinoma in‐vivo models have shown to have positive impact on disease outcomes and in preventing metastases.^104^ Survival benefit with antibiotic use has been shown in patient cohort receiving chemotherapy for metastatic pancreatic ductal adenocarcinoma.^105^ Currently, literature is unavailable for such clinical trials in HNSCC, however similar treatment strategies need to be tested in in‐vivo models, followed by its implementation in patient cohorts, in a tumour subsite‐specific manner. On the contrary, HPV‐positive oropharyngeal (OPSCC) and EBV‐positive nasopharyngeal tumours are proposed to show better prognosis, higher survival and respond to immunotherapeutic interventions due to their intra‐tumour immune composition.^106^, ^107^ The molecular profile of the HPV positive OPSCC also are anticipated to demand lower intensity adjuvant treatment, which is currently under scrutiny in a clinical trial (NCT02215265). Also, for EBV positive nasopharyngeal cancers therapeutic recombinant vaccine has been tested in a phase II clinical trial to treat patients with residual EBV DNA load after conventional therapy (NCT01094405).

Inversely, the microbiome affects the outcome of the therapy regimens in HNSCC patients. A detailed review cataloguing the clinical studies investigating associations between the HNSCC associated microbiome and their therapeutic implications has been presented elsewhere.^56^ Use of broad range antibiotics in patients with locally advanced head and neck cancer treated with chemo‐radiotherapy had negative impact on overall survival, progression‐free and disease‐specific survival.^108^ Another study reported enrichment of *Fusobacterium nucleatum* and *Mycoplasma* in patients with reduced sensitivity to induction chemotherapy, functionally through platinum drug resistance pathway in oral squamous cell carcinoma.^109^ Similarly, chemotherapy resistance due to abundance of opportunistic microbes is clearly demonstrated in colorectal carcinoma.^110^ Large HNSCC cohort studies screening for levels of the inflammatory bacterium and intervention with targeted antibiotics for site specific tumours are indispensable for development of novel treatment strategies to improve disease outcomes.

Large scale genomic studies have broadly classified the HNSCC tumours into distinct sub‐types mentioned above, including HPV positive, EBV positive and *Fusobacterium* (or infectious gram negative *oncobacterium*) positive tumours. Due to the influence of the specific microbes, these tumours also show a distinct immune profile which present novel targeting opportunities in some, whereas additional treatment challenges in others. Immune infiltration is closely linked to the therapy response in HNSCC, as well as other solid tumours.^111^, ^112^, ^113^ The immune profiles also have a very close association with the landscape of infectious pathogens present in the tumour microenvironment. Hence, its summary is equally important in this context. However, it is found to be out of scope for the current review and has been cursorily depicted in Figure 1 based on the following literature.^114^, ^115^, ^116^ In depth associations and interactions of genomic, microbial, and immune landscape of the tumours, will provide several additional answers on the way tumours respond to specific therapies, as well as point towards novel therapeutic approaches.

## 6 CONCLUSION

Microbial dysbiosis or pathogenic infection of specific microbes may greatly influence host genome and epigenome, leading to changes in gene expression and cellular processes that contribute to HNSCC pathogenesis. However, more research is needed to understand the complex interactions between host–microbe interactions in HNSCC pathogenesis, similar to colon cancer. Systematic analyses bridging gap between the pathogen induced molecular portraits of HNSCC and the clinical disease outcomes due to microbiome interference will pave way to novel treatment and preventive approaches.

## AUTHOR CONTRIBUTIONS

**Sanket Desai:** Conceptualization (lead); investigation (lead); writing – original draft (lead); writing – review and editing (lead).

## FUNDING INFORMATION

No funding was obtained for this work.

## CONFLICT OF INTEREST STATEMENT

The author declare that they have no competing interest.

## ETHICS STATEMENT

Not applicable.

## Data Availability

Data sharing is not applicable to this article as no new data were created or analyzed in this study.

## References

[1] Sung H , Ferlay J , Siegel RL , et al. Global cancer statistics 2020: GLOBOCAN estimates of incidence and mortality worldwide for 36 cancers in 185 countries. CA Cancer J Clin. 2021;71(3):209‐249. 33538338 10.3322/caac.21660

[2] Hashibe M , Hunt J , Wei M , Buys S , Gren L , Lee Y‐CA . Tobacco, alcohol, body mass index, physical activity, and the risk of head and neck cancer in the prostate, lung, colorectal, and ovarian (PLCO) cohort. Head Neck. 2013;35(7):914‐922. 22711227 10.1002/hed.23052

[3] Network TCGA . Comprehensive genomic characterization of head and neck squamous cell carcinomas. Nature. 2015;517(7536):576‐582. 25631445 10.1038/nature14129 PMC4311405

[4] Upadhyay P , Gardi N , Desai S , et al. Genomic characterization of tobacco/nut chewing HPV‐negative early stage tongue tumors identify MMP10 as a candidate to predict metastases. Oral Oncol. 2017;73:56‐64. 28939077 10.1016/j.oraloncology.2017.08.003 PMC5628952

[5] Ju Y , Wu X , Wang H , et al. Genomic landscape of head and neck squamous cell carcinoma across different anatomic sites in Chinese population. Front Genet. 2021;12:680699. 10.3389/fgene.2021.680699 PMC8236955 34194478

[6] Parkin DM . The global health burden of infection‐associated cancers in the year 2002. Int J Cancer. 2006;118(12):3030‐3044. 16404738 10.1002/ijc.21731

[7] de Martel C , Georges D , Bray F , Ferlay J , Clifford GM . Global burden of cancer attributable to infections in 2018: a worldwide incidence analysis. Lancet Glob Health. 2020;8(2):e180‐e190. 31862245 10.1016/S2214-109X(19)30488-7

[8] Krump NA , You J . Molecular mechanisms of viral oncogenesis in humans. Nat Rev Microbiol. 2018;16(11):684‐698. 30143749 10.1038/s41579-018-0064-6 PMC6336458

[9] Littman AJ , Jackson LA , Vaughan TL . Chlamydia pneumoniae and Lung cancer: epidemiologic evidence. Cancer Epidemiol Biomarkers Prev. 2005;14(4):773‐778. 15824142 10.1158/1055-9965.EPI-04-0599

[10] Mager DL . Bacteria and cancer: cause, coincidence or cure? A review. J Transl Med. 2006;4:14. 10.1186/1479-5876-4-14 PMC1479838 16566840

[11] Iyer P , Barreto SG , Sahoo B , et al. Non‐typhoidal Salmonella DNA traces in gallbladder cancer. Infect Agents Cancer. 2016;11(1):12. 10.1186/s13027-016-0057-x PMC4776363 26941832

[12] Ellmerich S , Scholler M , et al. Promotion of intestinal carcinogenesis by Streptococcus bovis. Carcinogenesis. 2000;21(4):753‐756. 10753212 10.1093/carcin/21.4.753

[13] Kostic AD , Gevers D , Pedamallu CS , et al. Genomic analysis identifies association of Fusobacterium with colorectal carcinoma. Genome Res. 2012;22(2):292‐298. 22009990 10.1101/gr.126573.111 PMC3266036

[14] Ulger Toprak N , Yagci A , Gulluoglu BM , et al. A possible role of Bacteroides fragilis enterotoxin in the aetiology of colorectal cancer. Clin Microbiol Infect. 2006;12(8):782‐786. 16842574 10.1111/j.1469-0691.2006.01494.x

[15] Swidsinski A , Khilkin M , Kerjaschki D , et al. Association between intraepithelial Escherichia coli and colorectal cancer. Gastroenterology. 1998;115(2):281‐286. 9679033 10.1016/s0016-5085(98)70194-5

[16] Castellarin M , Warren RL , Freeman JD , et al. Fusobacterium nucleatum infection is prevalent in human colorectal carcinoma. Genome Res. 2012;22(2):299‐306. 22009989 10.1101/gr.126516.111 PMC3266037

[17] Lamont RJ , Koo H , Hajishengallis G . The oral microbiota: dynamic communities and host interactions. Nat Rev Microbiol. 2018;16(12):745‐759. 30301974 10.1038/s41579-018-0089-x PMC6278837

[18] Rodríguez‐Lozano B , González‐Febles J , Garnier‐Rodríguez JL , et al. Association between severity of periodontitis and clinical activity in rheumatoid arthritis patients: a case–control study. Arthritis Res Ther. 2019;21(1):27. 30658685 10.1186/s13075-019-1808-z PMC6339403

[19] Liu X‐X , Jiao B , Liao X‐X , et al. Analysis of salivary microbiome in patients with Alzheimer's disease. J Alzheimers Dis. 2019;72(2):633‐640. 31594229 10.3233/JAD-190587

[20] Czesnikiewicz‐Guzik M , Osmenda G , Siedlinski M , et al. Causal association between periodontitis and hypertension: evidence from Mendelian randomization and a randomized controlled trial of non‐surgical periodontal therapy. Eur Heart J. 2019;40(42):3459‐3470. 31504461 10.1093/eurheartj/ehz646 PMC6837161

[21] Pessoa L , Aleti G , Choudhury S , et al. Host‐microbial interactions in systemic lupus erythematosus and periodontitis. Front Immunol. 2019;10:2602. 10.3389/fimmu.2019.02602 PMC6861327 31781106

[22] Flemer B , Warren RD , Barrett MP , et al. The oral microbiota in colorectal cancer is distinctive and predictive. Gut. 2018;67(8):1454‐1463. 28988196 10.1136/gutjnl-2017-314814 PMC6204958

[23] Fan X , Alekseyenko AV , Wu J , et al. Human oral microbiome and prospective risk for pancreatic cancer: a population‐based nested case‐control study. Gut. 2018;67(1):120‐127. 27742762 10.1136/gutjnl-2016-312580 PMC5607064

[24] Kawasaki M , Ikeda Y , Ikeda E , et al. Oral infectious bacteria in dental plaque and saliva as risk factors in patients with esophageal cancer. Cancer. 2020;127(4):512‐519. 33156979 10.1002/cncr.33316

[25] Shi J , Yang Y , Xie H , et al. Association of oral microbiota with lung cancer risk in a low‐income population in the southeastern USA. Cancer Causes Control. 2021;32(12):1423‐1432. 34432217 10.1007/s10552-021-01490-6 PMC8541916

[26] Vogtmann E , Hua X , Yu G , et al. The Oral microbiome and Lung cancer risk: an analysis of 3 prospective cohort studies. J Natl Cancer Inst. 2022;114(11):1501‐1510. 35929779 10.1093/jnci/djac149 PMC9664178

[27] Hayes RB , Ahn J , Fan X , et al. Association of Oral Microbiome with Risk for incident head and neck squamous cell cancer. JAMA Oncol. 2018;4(3):358. 29327043 10.1001/jamaoncol.2017.4777 PMC5885828

[28] Structure, function and diversity of the healthy human microbiome. Nature. 2012;486(7402):207‐214. 22699609 10.1038/nature11234 PMC3564958

[29] Caselli E , Fabbri C , D'Accolti M , et al. Defining the oral microbiome by whole‐genome sequencing and resistome analysis: the complexity of the healthy picture. BMC Microbiol. 2020;20(1):120. 32423437 10.1186/s12866-020-01801-y PMC7236360

[30] Bratman SV , Bruce JP , O'Sullivan B , et al. Human papillomavirus genotype association with survival in head and neck squamous cell carcinoma. JAMA Oncol. 2016;2(6):823. 27010835 10.1001/jamaoncol.2015.6587

[31] Narisawa‐Saito M , Kiyono T . Basic mechanisms of high‐risk human papillomavirus‐induced carcinogenesis: roles of E6 and E7 proteins. Cancer Sci. 2007;98(10):1505‐1511. 17645777 10.1111/j.1349-7006.2007.00546.x PMC11158331

[32] Koppad R , Suresh G , Prakash BV , Sabitha KS , Dhara PS . Prognostic indicators of oral squamous cell carcinoma. Ann Maxillofac Surg. 2019;9(2):364. 31909017 10.4103/ams.ams_253_18 PMC6933976

[33] Leemans CR , Snijders PJF , Brakenhoff RH . The molecular landscape of head and neck cancer. Nat Rev Cancer. 2018;18(5):269‐282. 29497144 10.1038/nrc.2018.11

[34] Fernandes Q , Merhi M , Raza A , et al. Role of Epstein–Barr virus in the pathogenesis of head and neck cancers and its potential as an immunotherapeutic target. Front Oncol. 2018;8:8. 30035101 10.3389/fonc.2018.00257 PMC6043647

[35] Tsang CM , Lui VWY , Bruce JP , Pugh TJ , Lo KW . Translational genomics of nasopharyngeal cancer. Semin Cancer Biol. 2020;61:84‐100. 31521748 10.1016/j.semcancer.2019.09.006

[36] Vasquez AA , Ram JL , Qazazi MS , Sun J , Kato I . Oral microbiome: potential link to systemic diseases and Oral cancer. Mechanisms underlying host‐microbiome interactions in pathophysiology of human diseases. Springer, Boston, MA, 2018:195‐246.

[37] Hooper SJ , Crean S‐J , Fardy MJ , et al. A molecular analysis of the bacteria present within oral squamous cell carcinoma. J Med Microbiol. 2007;56(12):1651‐1659. 18033835 10.1099/jmm.0.46918-0

[38] Frank DN , Qiu Y , Cao Y , et al. A dysbiotic microbiome promotes head and neck squamous cell carcinoma. Oncogene. 2022;41(9):1269‐1280. 35087236 10.1038/s41388-021-02137-1 PMC8882136

[39] Gong H , Shi Y , Xiao X , et al. Alterations of microbiota structure in the larynx relevant to laryngeal carcinoma. Sci Rep. 2017;7(1):5507. 28710395 10.1038/s41598-017-05576-7 PMC5511217

[40] Desai S , Dharavath B , Manavalan S , et al. Fusobacterium nucleatum is associated with inflammation and poor survival in early‐stage HPV‐negative tongue cancer. NAR Cancer. 2022;4(1):zcac006. 10.1093/narcan/zcac006 PMC8894079 35252868

[41] Ganly I , Yang L , Giese RA , et al. Periodontal pathogens are a risk factor of oral cavity squamous cell carcinoma, independent of tobacco and alcohol and human papillomavirus. Int J Cancer. 2019;145(3):775‐784. 30671943 10.1002/ijc.32152 PMC6554043

[42] Nagy KN , Sonkodi I , Szoke I , Nagy E , Newman HN . The microflora associated with human oral carcinomas. Oral Oncol. 1998;34(4):304‐308. 9813727

[43] Wen L , Mu W , Lu H , et al. Porphyromonas gingivalis promotes Oral squamous cell carcinoma progression in an immune microenvironment. J Dent Res. 2020;99(6):666‐675. 32298192 10.1177/0022034520909312

[44] Gallimidi AB , Fischman S , Revach B , et al. Periodontal pathogens Porphyromonas gingivalis and Fusobacterium nucleatum promote tumor progression in an oral‐specific chemical carcinogenesis model. Oncotarget. 2015;6(26):22613‐22623. 26158901 10.18632/oncotarget.4209 PMC4673186

[45] Yoshimura A , Kaneko T , Kato Y , Golenbock DT , Hara Y . Lipopolysaccharides from Periodontopathic BacteriaPorphyromonas gingivalisandCapnocytophaga ochraceaAre antagonists for human toll‐like receptor 4. Infect Immun. 2002;70(1):218‐225. 11748186 10.1128/IAI.70.1.218-225.2002 PMC127593

[46] Tsolis RM , Kamarajan P , Ateia I , et al. Periodontal pathogens promote cancer aggressivity via TLR/MyD88 triggered activation of integrin/FAK signaling that is therapeutically reversible by a probiotic bacteriocin. PLoS Pathog. 2020;16(10):e1008881. 10.1371/journal.ppat.1008881 PMC7529280 33002094

[47] Zhao H , Wu L , Yan G , et al. Inflammation and tumor progression: signaling pathways and targeted intervention. Signal Transduct Target Ther. 2021;6(1):263. 10.1038/s41392-021-00658-5 PMC8273155 34248142

[48] Inaba H , Sugita H , Kuboniwa M , et al. Porphyromonas gingivalispromotes invasion of oral squamous cell carcinoma through induction of proMMP9 and its activation. Cell Microbiol. 2014;16(1):131‐145. 23991831 10.1111/cmi.12211 PMC3939075

[49] Dharavath B , Butle A , Pal A , et al. Role of miR‐944/MMP10/AXL‐ axis in lymph node metastasis in tongue cancer. Commun Biol. 2023;6(1):57. 36650344 10.1038/s42003-023-04437-6 PMC9845355

[50] Singh S , Singh AK . Porphyromonas gingivalis in oral squamous cell carcinoma: a review. Microbes Infect. 2022;24(3):104925. 34883247 10.1016/j.micinf.2021.104925

[51] McIlvanna E , Linden GJ , Craig SG , Lundy FT , James JA . Fusobacterium nucleatum and oral cancer: a critical review. BMC Cancer. 2021;21(1):1212. 34774023 10.1186/s12885-021-08903-4 PMC8590362

[52] Dohlman AB , Arguijo Mendoza D , Ding S , et al. The cancer microbiome atlas: a pan‐cancer comparative analysis to distinguish tissue‐resident microbiota from contaminants. Cell Host Microbe. 2021;29(2):281‐98.e5. 33382980 10.1016/j.chom.2020.12.001 PMC7878430

[53] Chung L‐M , Liang J‐A , Lin C‐L , Sun L‐M , Kao C‐H . Cancer risk in patients with candidiasis: a nationwide population‐based cohort study. Oncotarget. 2017;8(38):63562‐63573. 28969011 10.18632/oncotarget.18855 PMC5609943

[54] Narunsky‐Haziza L , Sepich‐Poore GD , Livyatan I , et al. Pan‐cancer analyses reveal cancer‐type‐specific fungal ecologies and bacteriome interactions. Cell. 2022;185(20):3789‐806.e17. 36179670 10.1016/j.cell.2022.09.005 PMC9567272

[55] Xue C , Chu Q , Zheng Q , et al. Current understanding of the intratumoral microbiome in various tumors. Cell Rep Med. 2023;4(1):100884. 36652905 10.1016/j.xcrm.2022.100884 PMC9873978

[56] Reis Ferreira M , Pasto A , Ng T , et al. The microbiota and radiotherapy for head and neck cancer: what should clinical oncologists know? Cancer Treat Rev. 2022;109:102442. 35932549 10.1016/j.ctrv.2022.102442

[57] Hayes DN , Van Waes C , Seiwert TY . Genetic landscape of human papillomavirus–associated head and neck cancer and comparison to tobacco‐related tumors. J Clin Oncol. 2015;33(29):3227‐3234. 26351353 10.1200/JCO.2015.62.1086 PMC4586167

[58] Rampias T , Sasaki C , Weinberger P , Psyrri A . E6 and E7 gene silencing and transformed phenotype of human papillomavirus 16‐positive oropharyngeal cancer cells. J Natl Cancer Inst. 2009;101(6):412‐423. 19276448 10.1093/jnci/djp017

[59] Henderson S , Chakravarthy A , Su X , Boshoff C , Fenton TR . APOBEC‐mediated cytosine deamination links PIK3CA helical domain mutations to human papillomavirus‐driven tumor development. Cell Rep. 2014;7(6):1833‐1841. 24910434 10.1016/j.celrep.2014.05.012

[60] Kondo S , Wakae K , Wakisaka N , et al. APOBEC3A associates with human papillomavirus genome integration in oropharyngeal cancers. Oncogene. 2016;36(12):1687‐1697. 27694899 10.1038/onc.2016.335

[61] Koneva LA , Zhang Y , Virani S , et al. HPV integration in HNSCC correlates with survival outcomes, immune response signatures, and candidate drivers. Mol Cancer Res. 2018;16(1):90‐102. 28928286 10.1158/1541-7786.MCR-17-0153 PMC5752568

[62] Lin D‐C , Meng X , Hazawa M , et al. The genomic landscape of nasopharyngeal carcinoma. Nat Genet. 2014;46(8):866‐871. 24952746 10.1038/ng.3006

[63] Ernberg IT , Deng W , Pang PS , et al. Epstein‐Barr virus‐encoded latent membrane protein 1 impairs G2 checkpoint in human nasopharyngeal epithelial cells through defective Chk1 activation. PLoS One. 2012;7(6):e39095. 10.1371/journal.pone.0039095 PMC3382577 22761726

[64] Leong MML , Cheung AKL , Dai W , et al. EBV infection is associated with histone bivalent switch modifications in squamous epithelial cells. Proc Natl Acad Sci. 2019;116(28):14144‐14153. 31235597 10.1073/pnas.1821752116 PMC6628793

[65] Beyaert S , Machiels J‐P , Schmitz S . Vaccine‐based immunotherapy for head and neck cancers. Cancer. 2021;13(23):6041. 10.3390/cancers13236041 PMC8656843 34885150

[66] Schneider K , Grønhøj C , Hahn CH , von Buchwald C . Therapeutic human papillomavirus vaccines in head and neck cancer: a systematic review of current clinical trials. Vaccine. 2018;36(45):6594‐6605. 30268734 10.1016/j.vaccine.2018.09.027

[67] Machado AMD , Figueiredo C , Seruca R , Rasmussen LJ . Helicobacter pylori infection generates genetic instability in gastric cells. Biochim Biophys Acta. 2010;1806(1):58‐65. 20122996 10.1016/j.bbcan.2010.01.007

[68] Nougayrède J‐P , Homburg S , Fdr T , et al. Escherichia coli induces DNA double‐Strand breaks in eukaryotic cells. Science. 2006;313(5788):848‐851. 16902142 10.1126/science.1127059

[69] Goodwin AC , Shields CED , Wu S , et al. Polyamine catabolism contributes to enterotoxigenic Bacteroides fragilis‐induced colon tumorigenesis. Proc Natl Acad Sci. 2011;108(37):15354‐15359. 21876161 10.1073/pnas.1010203108 PMC3174648

[70] Hale VL , Jeraldo P , Chen J , et al. Distinct microbes, metabolites, and ecologies define the microbiome in deficient and proficient mismatch repair colorectal cancers. Genome Med. 2018;10(1):78. 30376889 10.1186/s13073-018-0586-6 PMC6208080

[71] Okita Y , Koi M , Takeda K , et al. Fusobacterium nucleatum infection correlates with two types of microsatellite alterations in colorectal cancer and triggers DNA damage. Gut Pathog. 2020;12(1):46. 10.1186/s13099-020-00384-3 PMC7526104 33005238

[72] Harrandah AM , Chukkapalli SS , Bhattacharyya I , Progulske‐Fox A , Chan EKL . Fusobacteria modulate oral carcinogenesis and promote cancer progression. J Oral Microbiol. 2020;13(1):1849493. 10.1080/20002297.2020.1849493 PMC7717872 33391626

[73] Kay J , Thadhani E , Samson L , Engelward B . Inflammation‐induced DNA damage, mutations and cancer. DNA Repair. 2019;83:102673. 31387777 10.1016/j.dnarep.2019.102673 PMC6801086

[74] Geng F , Zhang Y , Lu Z , Zhang S , Pan Y . Fusobacterium nucleatumCaused DNA damage and promoted cell proliferation by theKu70/p53Pathway in Oral cancer cells. DNA Cell Biol. 2020;39(1):144‐151. 31765243 10.1089/dna.2019.5064 PMC6978777

[75] Jin B , Robertson KD . DNA Methyltransferases, DNA damage repair, and cancer. Epigenetic alterations in Oncogenesis. Adv Exp Med Biol. 2013;754:3‐29. 10.1007/978-1-4419-9967-2_1 PMC3707278 22956494

[76] Suzuki T , Yamazaki H , Honda K , et al. Altered DNA methylation is associated with aberrant stemness gene expression in early‐stage HNSCC. Int J Oncol. 2019;55:915‐924. 31432153 10.3892/ijo.2019.4857

[77] Demokan S , Dalay N . Role of DNA methylation in head and neck cancer. Clin Epigenetics. 2011;2(2):123‐150. 22704334 10.1007/s13148-011-0045-3 PMC3365391

[78] Chen D , Wang M , Guo Y , et al. An aberrant DNA methylation signature for predicting the prognosis of head and neck squamous cell carcinoma. Cancer Med. 2021;10(17):5936‐5947. 34313009 10.1002/cam4.4142 PMC8419750

[79] Smith IM , Mydlarz WK , Mithani SK , Califano JA . DNA global hypomethylation in squamous cell head and neck cancer associated with smoking, alcohol consumption and stage. Int J Cancer. 2007;121(8):1724‐1728. 17582607 10.1002/ijc.22889

[80] Li HP , Leu YW , Chang YS . Epigenetic changes in virus‐associated human cancers. Cell Res. 2005;15(4):262‐271. 15857581 10.1038/sj.cr.7290295

[81] Matsusaka K , Kaneda A , Nagae G , et al. Classification of Epstein–Barr virus–positive gastric cancers by definition of DNA methylation Epigenotypes. Cancer Res. 2011;71(23):7187‐7197. 21990320 10.1158/0008-5472.CAN-11-1349

[82] Ally A , Balasundaram M , Carlsen R , et al. Comprehensive and integrative genomic characterization of hepatocellular carcinoma. Cell. 2017;169(7):1327‐41.e23. 28622513 10.1016/j.cell.2017.05.046 PMC5680778

[83] Vellano CP , Wentzensen N , Ojesina AI . Integrated genomic and molecular characterization of cervical cancer. Nature. 2017;543(7645):378‐384. 28112728 10.1038/nature21386 PMC5354998

[84] Lo KW , Cheung ST , Leung SF , et al. Hypermethylation of the p16 gene in nasopharyngeal carcinoma. Cancer Res. 1996;56(12):2721‐2725. 8665502

[85] Hino R , Uozaki H , Murakami N , et al. Activation of DNA methyltransferase 1 by EBV latent membrane protein 2A leads to promoter hypermethylation of PTEN gene in gastric carcinoma. Cancer Res. 2009;69(7):2766‐2774. 19339266 10.1158/0008-5472.CAN-08-3070

[86] Yeung CLA , Tsang WP , Tsang TY , Co NN , Yau PL , Kwok TT . HPV‐16 E6 upregulation of DNMT1 through repression of tumor suppressor p53. Oncol Rep. 2010;24(6):1599–604. 10.3892/or_00001023 21042757

[87] Pan X , Gong D , Nguyen DN , et al. Early microbial colonization affects DNA methylation of genes related to intestinal immunity and metabolism in preterm pigs. DNA Res. 2018;25(3):287‐296. 29365082 10.1093/dnares/dsy001 PMC6014285

[88] Paul B , Barnes S , Demark‐Wahnefried W , et al. Influences of diet and the gut microbiome on epigenetic modulation in cancer and other diseases. Clin Epigenetics. 2015;7(1):112. 10.1186/s13148-015-0144-7 PMC4609101 26478753

[89] Miro‐Blanch J , Yanes O . Epigenetic regulation at the interplay between gut microbiota and host metabolism. Front Genet. 2019;10:638. 10.3389/fgene.2019.00638 PMC6628876 31338107

[90] Mirzaei R , Afaghi A , Babakhani S , et al. Role of microbiota‐derived short‐chain fatty acids in cancer development and prevention. Biomed Pharmacother. 2021;139:111619. 33906079 10.1016/j.biopha.2021.111619

[91] Sobhani I , Rotkopf H , Khazaie K . Bacteria‐related changes in host DNA methylation and the risk for CRC. Gut Microbes. 2020;12(1):1800898. 32931352 10.1080/19490976.2020.1800898 PMC7575230

[92] Xia X , Wu WKK , Wong SH , et al. Bacteria pathogens drive host colonic epithelial cell promoter hypermethylation of tumor suppressor genes in colorectal cancer. Microbiome. 2020;8(1):108. 32678024 10.1186/s40168-020-00847-4 PMC7367367

[93] Zhang H , Ma RR , Wang XJ , et al. KIF26B, a novel oncogene, promotes proliferation and metastasis by activating the VEGF pathway in gastric cancer. Oncogene. 2017;36(40):5609‐5619. 28581513 10.1038/onc.2017.163

[94] Wang J , Cui F , Wang X , et al. Elevated kinesin family member 26B is a prognostic biomarker and a potential therapeutic target for colorectal cancer. J Exp Clin Cancer Res. 2015;34(1):13. 25652119 10.1186/s13046-015-0129-6 PMC4322797

[95] Chen S , Zhang L , Li M , et al. Fusobacterium nucleatum reduces METTL3‐mediated m6A modification and contributes to colorectal cancer metastasis. Nat Commun. 2022;13(1):1248. 10.1038/s41467-022-28913-5 PMC8913623 35273176

[96] Chen Z , Wong PY , Ng CWK , et al. The intersection between Oral microbiota, host gene methylation and patient outcomes in head and neck squamous cell carcinoma. Cancer. 2020;12(11):3425. 10.3390/cancers12113425 PMC7698865 33218162

[97] Martins MD , Jiao Y , Larsson L , et al. Epigenetic modifications of histones in periodontal disease. J Dent Res. 2015;95(2):215‐222. 26496800 10.1177/0022034515611876

[98] Wang L , Zhang W , Wu X , et al. MIAOME: human microbiome affect the host epigenome. Comput Struct Biotechnol J. 2022;20:2455‐2463. 35664224 10.1016/j.csbj.2022.05.024 PMC9136154

[99] Zhou X , Hao Y , Peng X , et al. The clinical potential of Oral microbiota as a screening tool for Oral squamous cell carcinomas. Front Cell Infect Microbiol. 2021;11:11. 10.3389/fcimb.2021.728933 PMC8416267 34485181

[100] Kong C . Human oral microbiome dysbiosis as a novel tool for detecting noninvasive biomarkers for colorectal cancer. J Clin Oncol. 2021;39(3_suppl):23.

[101] Sun H , Zhao X , Zhou Y , et al. Characterization of Oral microbiome and exploration of potential biomarkers in patients with pancreatic cancer. Biomed Res Int. 2020;2020:1‐11. 10.1155/2020/4712498 PMC7652608 33204698

[102] Ahn J , Segers S , Hayes RB . Periodontal disease, Porphyromonas gingivalis serum antibody levels and orodigestive cancer mortality. Carcinogenesis. 2012;33(5):1055‐1058. 22367402 10.1093/carcin/bgs112 PMC3334514

[103] Mima K , Nishihara R , Qian ZR , et al. Fusobacterium nucleatumin colorectal carcinoma tissue and patient prognosis. Gut. 2016;65(12):1973‐1980. 26311717 10.1136/gutjnl-2015-310101 PMC4769120

[104] Bullman S , Pedamallu CS , Sicinska E , et al. Analysis of Fusobacterium persistence and antibiotic response in colorectal cancer. Science. 2017;358(6369):1443‐1448. 29170280 10.1126/science.aal5240 PMC5823247

[105] Fulop DJ , Zylberberg HM , Wu YL , et al. Association of Antibiotic Receipt with Survival among Patients with Metastatic Pancreatic Ductal Adenocarcinoma Receiving Chemotherapy. JAMA Netw Open. 2023;6(3):e234254. 36951863 10.1001/jamanetworkopen.2023.4254 PMC10037151

[106] Julian R , Savani M , Bauman JE . Immunotherapy approaches in HPV‐associated head and neck cancer. Cancer. 2021;13(23):5889. 10.3390/cancers13235889 PMC8656769 34884999

[107] Hong M , Tang K , Qian J , et al. Immunotherapy for EBV‐associated nasopharyngeal carcinoma. Crit Rev Oncog. 2018;23(3–4):219‐234. 30311576 10.1615/CritRevOncog.2018027528

[108] Nenclares P , Bhide SA , Sandoval‐Insausti H , et al. Impact of antibiotic use during curative treatment of locally advanced head and neck cancers with chemotherapy and radiotherapy. Eur J Cancer. 2020;131:9‐15. 32248073 10.1016/j.ejca.2020.02.047

[109] Rui M , Zhang X , Huang J , et al. The baseline oral microbiota predicts the response of locally advanced oral squamous cell carcinoma patients to induction chemotherapy: a prospective longitudinal study. Radiother Oncol. 2021;164:83‐91. 34571091 10.1016/j.radonc.2021.09.013

[110] Pandey K , Umar S . Microbiome in drug resistance to colon cancer. Curr Opin Phys Ther. 2021;23:23. 10.1016/j.cophys.2021.100472 PMC8425411 34514218

[111] Mandal R , Şenbabaoğlu Y , Desrichard A , et al. The head and neck cancer immune landscape and its immunotherapeutic implications. JCI Insight. 2016;1(17):e89829. 10.1172/jci.insight.89829 PMC5070962 27777979

[112] Economopoulou P , Perisanidis C , Giotakis EI , Psyrri A . The emerging role of immunotherapy in head and neck squamous cell carcinoma (HNSCC): anti‐tumor immunity and clinical applications. Ann Transl Med. 2016;4(9):173. 27275486 10.21037/atm.2016.03.34 PMC4876265

[113] Datta M , Coussens LM , Nishikawa H , Hodi FS , Jain RK . Reprogramming the tumor microenvironment to improve immunotherapy: emerging strategies and combination therapies. Am Soc Clin Oncol Educ Book. 2019;39:165‐174. 31099649 10.1200/EDBK_237987 PMC6596289

[114] Chen YP , Wang YQ , Lv JW , et al. Identification and validation of novel microenvironment‐based immune molecular subgroups of head and neck squamous cell carcinoma: implications for immunotherapy. Ann Oncol. 2019;30(1):68‐75. 30407504 10.1093/annonc/mdy470

[115] Varn FS , Schaafsma E , Wang Y , Cheng C . Genomic characterization of six virus‐associated cancers identifies changes in the tumor immune microenvironment and altered genetic programs. Cancer Res. 2018;78(22):6413‐6423. 30254145 10.1158/0008-5472.CAN-18-1342 PMC6239894

[116] Johnson DE , Burtness B , Leemans CR , Lui VWY , Bauman JE , Grandis JR . Head and neck squamous cell carcinoma. Nat Rev Dis Primers. 2020;6(1):92. 33243986 10.1038/s41572-020-00224-3 PMC7944998

